# The incidence of pelvic fractures and related surgery in the Finnish adult population: a nationwide study of 33,469 patients between 1997 and 2014

**DOI:** 10.1080/17453674.2020.1771827

**Published:** 2020-06-05

**Authors:** Pasi P Rinne, Minna K Laitinen, Pekka Kannus, Ville M Mattila

**Affiliations:** a Vaasa Central Hospital, Vaasa, Finland;; b Helsinki University Hospital, Helsinki, Finland;; c University of Helsinki, Helsinki, Finland;; d School of Medicine, Tampere University, Tampere, Finland;; e Department of Orthopaedics, Unit of Musculoskeletal Surgery, Tampere University Hospital, Tampere, Finland

## Abstract

Background and purpose — Information on the epidemiological trends of pelvic fractures and fracture surgery in the general population is limited. We therefore determined the incidence of pelvic fractures in the Finnish adult population between 1997 and 2014 and assessed the incidence and trends of fracture surgery.

Patients and methods — We used data from the Finnish National Discharge Register (NHDR) to calculate the incidence of pelvic fractures and fracture surgery. All patients 18 years of age or older were included in the study. The NHDR covers the whole Finnish population and gives information on health care services and the surgical procedures performed.

Results and interpretation — We found that in Finnish adults the overall incidence of hospitalization for a pelvic fracture increased from 34 to 56/100,000 person-years between 1997 and 2014. This increase was most apparent for the low-energy fragility fractures of the elderly female population. The ageing of the population is likely therefore to partly explain this increase. The annual number and incidence of pelvic fracture surgery also rose between 1997 and 2014, from 118 (number) and 3.0 (incidence) in 1997 to 187 and 4.3 in 2014, respectively. The increasing number and incidence of pelvic fractures in the elderly population will increase the need for social and healthcare services. The main focus should be on fracture prevention.

Pelvic fractures range from minor to major trauma and constitute about 3% to 8% of all fractures treated in hospitals (Court-Brown and Caesar 2006). The incidence of pelvic fractures has varied from 17 to 364/100,000 person-years (Melton et al. 1981, Ragnarsson and Jacobsson 1992, Lüthje et al. 1995, Kannus et al. 2000, Balogh et al. 2007, Andrich et al. 2015, Kannus et al. 2015, Verbeek et al. 2017). This wide range in incidence rates can be explained by different study populations with varying age, and by variations in study designs and follow-up periods. In previous studies, the incidence (n/100,000 person-years) of pelvic fractures was in the United States 37 between 1968 and 1977 (Melton et al. 1981), in Sweden 20 between 1976 and 1985 (Ragnarsson and Jacobsson 1992), in Finland 24 in 1988 (Lüthje et al. 1995), in the Finnish population aged 60 years or older 20 in 1970 and 92 in 1997 (Kannus et al. 2000), in Australia 23 between 2005 and 2006 (Balogh et al. 2007), in the German population aged 60 years or older 22 between 2008 and 2011 (Andrich et al. 2015), in the Finnish population aged 80 years or older 73 in 1971 and 364 in 2013 (Kannus et al. 2015) and in the Netherlands 14 between 2008 and 2012 (Verbeek et al. 2017).

In the 80 years and older population, the incidence of low-energy pelvic fractures seems to be increasing (Kannus et al. 2015). Indeed, between 1997 and 2014, the incidence of acetabular fractures, especially low-energy acetabular fractures, rose in Finland (Rinne et al. 2017), whereas the incidence of high-energy acetabular fractures remained at the same level. Notably, since 1997, the incidence of many other fall-related low-energy fractures, such as hip fractures, has decreased in Finland (Korhonen et al. 2013, Kannus et al. 2018).

Most pelvic fracture studies concentrate on surgical treatment, even though the majority of these fractures can be treated nonoperatively (Osterhoff et al. 2019, Tornetta et al. 2019). Unstable and dislocated pelvic fractures often need surgery, while stable, non-displaced, or minimally displaced fractures, mostly occurring in elderly people after a simple fall, can usually be treated nonsurgically. At present, however, there is only limited information available regarding the incidence and trends of pelvic fracture surgery in the general population.

We assessed the incidence of pelvic fractures in the Finnish adult population between 1997 and 2014 and the incidence and trends of pelvic fracture surgery.

## Patients and methods

The Finnish National Hospital Discharge Register (NHDR) (THL 2015) is maintained by the National Institute for Health and Welfare, which, in turn, is a research and development institute under the Finnish Ministry of Social Affairs and Health, Helsinki, Finland. The main purpose of the NHDR is to collect data on patients and hospitalization events in Finland. The NHDR covers the entire well-defined Finnish population of 5.5 million (in 2014) people (Statistics Finland 2019). The production of this NHDR information is mandatory for all medical service providers in Finland, and the funding of these institutions is based on this information.

The Finnish NHDR contains data on age, sex, domicile of the patient, length of hospital stay, primary and secondary diagnoses, surgical procedures performed during the stay, and trauma mechanisms. Since 1996, the diagnoses have been coded according to the 10th revision of the International Classification of Diseases (ICD) (World Health Organization 2004). The surgical procedures are coded according to the Finnish Classification of Procedures (FCP), which is based on the Nordic Classification of Surgical Procedures (NCSP) (Committee NM-S 2011, Lehtonen 2013). Data from the NHDR were available until 2014.

### Outcome variables

The main outcome variable for this study was the number of hospitalized patients with a primary or secondary diagnosis of pelvic fracture (ICD-10 codes S32.1, S32.2, S32.3, S32.4, S32.5, S32.7, or S32.8) in Finland between 1997 and 2014. All patients aged 18 years or older were included.

A secondary outcome variable was the number of surgical operations performed due to a pelvic fracture in Finland between 1997 and 2014 (FCP codes NEJ50, NEJ60, NEJ70, and NEJ86).

### Study population

**Figure 1.**
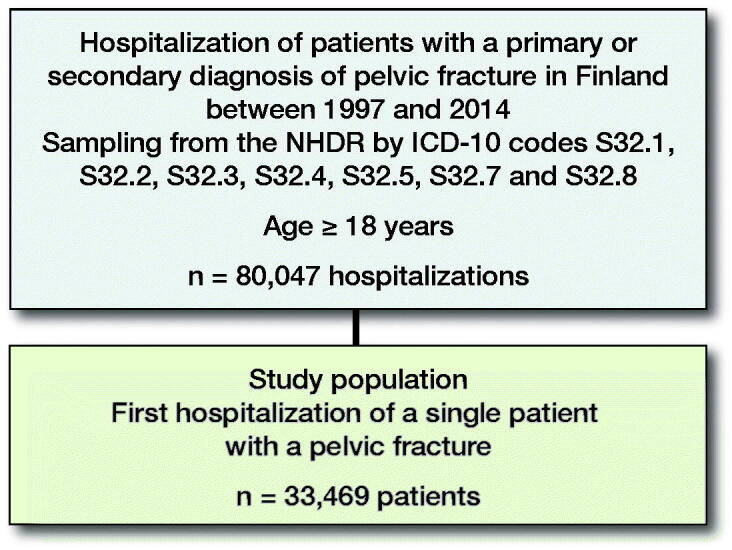
Flowchart of the study population.

All persons aged 18 years and older who were admitted to a hospital in Finland due to a pelvic fracture between 1997 and 2014 were included (Figure 1). The study population was categorized into 2 age groups: younger patients including adults under the general retirement age (18–64 years) and elderly patients over the general retirement age (65 years and older).

### Statistics

All incidences were calculated and expressed annually. To compute the incidence ratios of pelvic fractures leading to hospitalization, the annual mid-populations were obtained from Official Statistics of Finland (Statistics Finland 2019), a computer-based national population register. Crude incidence (later called “incidence”) and the age-standardized incidence of pelvic fractures were calculated for both sexes and were expressed as number of cases per 100,000 person-years. In the calculation of the age-standardized incidence rates, age adjustment was carried out by direct standardization using the mean population of Finland between 1997 and 2014 as the standard population. For the entire study period, 1 person was counted only once. The number of pelvic fracture operations were calculated for the study population.

The number and incidence of fractures were calculated for the entire Finnish adult population (3,988,773 adult inhabitants in 1997, and 4,396,261 in 2014) (Statistics Finland 2019) and expressed by sex and the two age categories (18–64 years and 65 and older). 95% confidence intervals (CI) were calculated for the incidence numbers.

### Ethics, funding, and potential conflicts of interest

In Finland—by law—register studies without the use of biological material do not require ethics committee approval. RECORD guidelines were followed (Benchimol et al. 2015). This research did not receive any funding. None of the authors has any conflicts of interest to declare. Permission to use the NHDR data for this study (THL/1244/5.05.00/2016) was provided by the National Institute for Health and Welfare, Helsinki, Finland.

## Results

### Pelvic fracture incidence

In the following, “incidence” refers to n/100,000 person-years.

**Figure 2.**
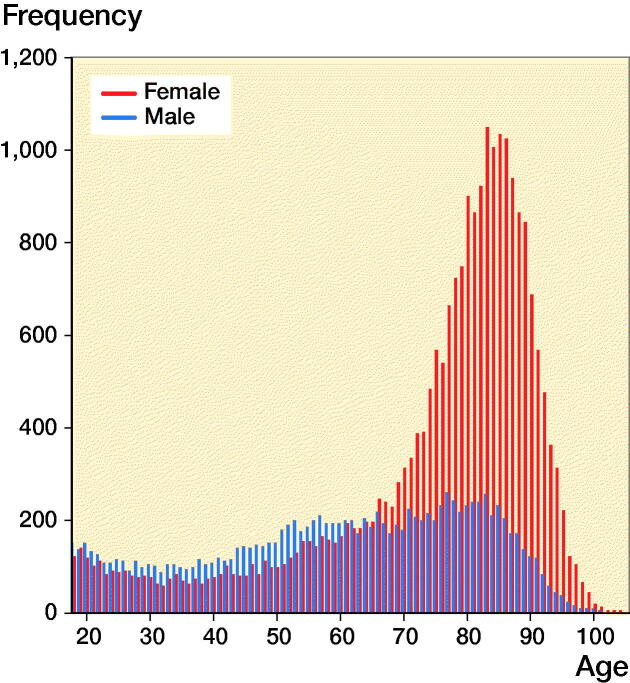
Age and sex distribution of patients with pelvic fracture in Finland from 1997 to 2014.

Between 1997 and 2014, there were 80,047 hospitalizations in Finland with a pelvic fracture diagnosis. In the case of multiple hospitalizations of a single patient, only the first episode with pelvic fracture diagnosis during the study period was included. Thus, 33,469 patients with pelvic fracture were included in the analysis. 2,755 surgical procedures for pelvic fractures were performed during this time period, which includes 8.2% of the fractures. The annual number of new pelvic fracture hospitalizations was 1,345 in 1997 and 2,460 in 2014. The age distribution of pelvic fracture patients was clearly bimodal: the major mode comprised older patients and the minor mode comprised younger patients (Figure 2).

**Figure 3.**
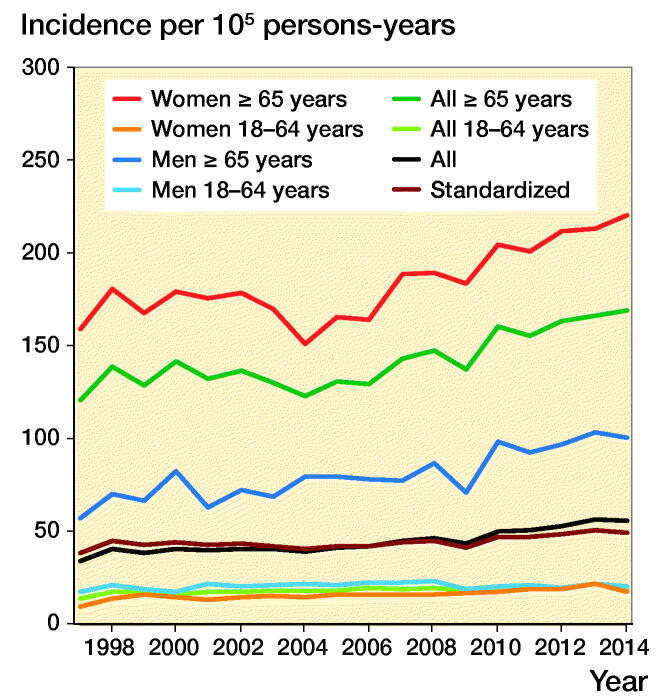
Incidence of pelvic fractures in Finland from 1997 to 2014.

The incidence of pelvic fracture hospitalization increased from 34 (CI 32–36) to 56 (CI 54–58) between 1997 and 2014 (Figure 3, Table). The age-standardized incidence increased correspondingly from 38 to 49.

### Sex and age

The frequency and incidence of pelvic fractures was different between the sexes, and this difference was more evident in the elderly population. Of all pelvic fractures, 66% occurred in women, and 52% of fractures occurred in women aged 65 years or older. 68% of all pelvic fractures were in the elderly population.

The age-specific incidence increased from 121 (CI 113–129) to 169 (CI 161–177) between 1997 and 2014 in persons aged 65 years and older. The incidence in the elderly female population was twice as high as that in the elderly male population. From 1997 to 2014, the incidence increased from 159 (CI 148–171) to 220 (CI 209–232) in the elderly female population and from 57 (CI 49–67) to 100 (CI 92–110) in the elderly male population. In the younger population, including both sexes, there was only a slight increase in the incidence of pelvic fractures from 14 (CI 12–15) to 19 (CI 17–20). The sex-specific increase in incidence in the younger population was from 17 (CI 15–19) to 20 (CI 18–22) in males and from 10 (CI 8–11) to 17 (CI 15–19) in females during the same period.

### Pelvic fracture surgery

**Figure 4.**
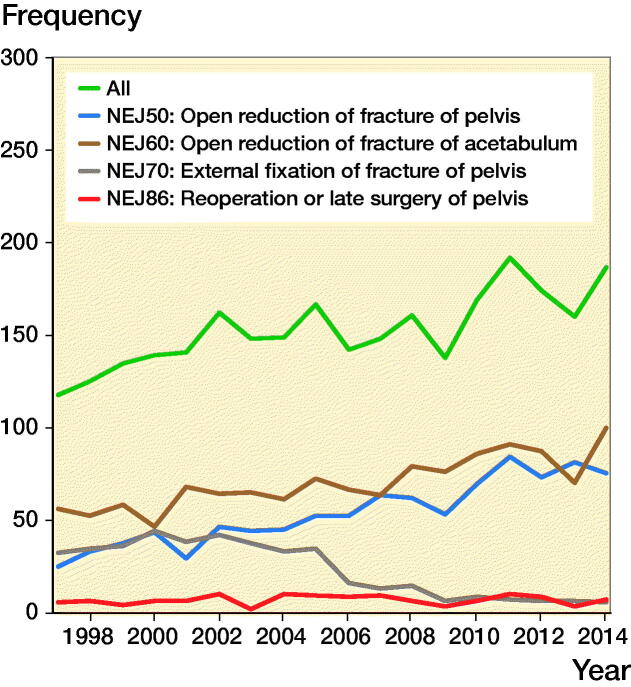
Number of surgical treatments due to a pelvic fracture in Finland from 1997 to 2014.

2,755 operations for pelvic fractures (8.2% of all pelvic fractures) were performed between the years 1997 and 2014. During the study period, the annual number and incidence of pelvic fracture operations (FCP classification codes NEJ50, NEJ60, NEJ70, and NEJ86 increased from 118 to 187 operations/year and from 3.0 to 4.3 operations (Figure 4).

The annual number and incidence of internal pelvic fracture fixations (NEJ50 or NEJ86) in both age groups and sexes increased from 30 to 82 operations/year and from 0.8 to 1.9 between 1997 and 2014. The annual number and incidence of internal pelvic fracture fixations was most common in young male patients (from 26 to 32 operations/year and from 1.6 to 1.9). In the younger female population, the number and incidence of internal pelvic fracture fixations was also increasing (from 2 to 16 operations/year and from 0.1 to 1.0). Between 1997 and 2014, the number and incidence of internal pelvic fracture fixations also increased in the elderly population from 2 to 34 operations/year and from 0.3 to 3.1. In the elderly male population, the number and incidence rose from 1 to 10 and from 0.4 to 2.1. In the elderly female population, the number and incidence rose from 1 to 24 operations/year and from 0.2 to 3.9.

The annual number and incidence of acetabular fracture operations (NEJ60) increased from 56 to 100 operation and from 1.4 to 2.3 from 1997 to 2014. In the elderly population, the increase was more evident; the annual number of surgeries increased from 15 to 55 operations/year and the incidence of operations from 2 to 5, respectively. In the younger population, the number and incidence of acetabular fracture surgery remained at the same level (from 41 to 45/year and from 1.3 to 1.4) from 1997 to 2014.

The rate and incidence of external fixation (NEJ70) operations was highest between the years 2000 and 2002 (range 44–42 operations/year, incidence range 1.1–1.0). However, since then, the number of external fixation decreased dramatically. The annual number of external fixations decreased from 32 to 5 operations/year and the incidence of the operations decreased from 0.8 to 0.1 between 1997 and 2014. External fixation was more common in the treatment of pelvic fractures in the younger population. In the elderly population, the number of external fixations has always been low, and no significant changes occurred during the study period. The use of external fixation became sporadic at the end of the study period. 

## Discussion

### Pelvic fracture incidence

The main finding in this nationwide study was that the overall incidence of pelvic fractures in adult Finns increased by 67% between 1997 and 2014. Pelvic fractures are common in high-energy-induced polytrauma patients. However, they are seen more frequently in elderly populations after a simple fall at ground level (Kannus et al. 2000, Rinne et al. 2017). Young adults are more at risk for high-energy pelvic fractures, whereas low-energy pelvic fractures mostly occur in elderly patients (Ragnarsson and Jacobsson 1992, Balogh et al. 2007, Andrich et al. 2017).

Our results also show that both the number and the increase in incidence of pelvic fractures were highest in the elderly female population, the latter rising from 159 to 221/100,000 person-years. Verbeek et al. reported similar findings in the Dutch population (Verbeek et al. 2017). In our elderly male population, there was also a substantial increase in pelvic fracture incidence from 57 to 101/100,000 person-years during the study period, although the incidence was lower than that in females.

The exact reasons for the rise in the elderly population’s and especially elderly female population’s age-specific incidence are unclear. The comparison between the crude incidence rate and age-standardized incidence rate shows that the changed age distribution does not explain all of the increase in incidence. Pelvic fractures in the elderly population are mostly low-energy fragility fractures that are related to falling and osteoporosis. The age structure of the Finnish population is changing and the mean age of the population is becoming older: the mean life expectancy in Finland was rising constantly during the study period (Statistics Finland 2017). People in Finland are also living longer at home. Impaired muscle strength, balance problems, physical inactivity, and degenerative joint diseases are common in the elderly population, and increase the risk of falling. Osteoporosis increases the fracture risk when falling. As pelvic fractures occur more frequently in the growing elderly population, the increase in the number and the incidence of fracture is expected to keep on rising.

The incidence of pelvic fractures in elderly people in Finland has been increasing for decades (Kannus et al. 2000) and is still increasing. Notably, since 1997, the crude incidence of hip fractures in elderly people in Finland has decreased, whereas it had been increasing for decades (Kannus et al. 2018). Kannus et al. (2000) showed that the incidence of hip fractures in the population aged 50 years or older in Finland increased considerably between 1970 and 1997 (from 160 to 440 fractures/100,000 person-years). Since then, however, the trend has been declining (340 fractures/100,000 person-years in 2015) (Kannus et al. 2006, Korhonen et al. 2013, Kannus et al. 2018). It might be suggested that a low-level fall in the elderly population would nowadays result more frequently in a fragility pelvic fracture instead of a hip fracture and this could partially explain the increase in incidence of pelvic fractures (Sullivan et al. 2014). Kannus et al. in their study in 2000 made a prediction for the annual number of first osteoporotic pelvic fractures in Finland to be about 1,400 by the year 2010 in the population aged 60 or older. In our present study, the number of pelvic fractures in the population aged 65 or older was 1,508 in the year 2010. Thus, the rate of the incidence of pelvic fractures in the elderly population was even higher than expected. The increasing number of pelvic fragility fractures cause challenges for the health and social care systems to be prepared and provide care and help for a rising number of fragility fracture victims. The main focus should be on fracture prevention by minimizing the risk factors of elderly people’s falls.

In our study, the overall incidence of pelvic fractures in adults was higher than previously presented (Melton et al. 1981, Ragnarsson and Jacobsson 1992, Lüthje et al. 1995, Balogh et al. 2007, Andrich et al. 2015, Verbeek et al. 2017). Based on the study by Lüthje et al., the incidence of pelvic fracture hospitalization in Finland was 24/100,000 person-years in 1988. In a recent study from the Netherlands, the average annual incidence of pelvic fractures was 14/100,000 person-years between 2008 and 2012 (Verbeek et al. 2017). In Sweden, the overall incidence of pelvic fractures requiring hospitalization was 20/100 et al. 000 person-years between 1976 and 1985 (Ragnarsson and Jacobsson 1992). The different incidence rates in previous studies (Melton et al. 1981, Ragnarsson and Jacobsson 1992, Lüthje et al. 1995, Kannus et al. 2000, Balogh et al. 2007, Andrich et al. 2015, Kannus et al. 2015, Verbeek et al. 2017) may have been caused by different study populations, variation in treatment protocols, or selection bias. The accuracy and coverage of a trauma registry of a trauma center is based on reported patients covered by the registry. As pelvic fragility fractures are often treated outside trauma centers, the number of patients with pelvic fragility fractures reported in a trauma register might be underestimated. In addition, health insurance registry-based study populations might differ from the general population in socio-economic or occupational status (Andrich et al. 2015). In nation-to-nation comparisons, the levels of the age-specific incidences of pelvic fracture depend much on selection of the age groups for each study. Also, the age structure of the background population varies. Thus, it is often difficult to compare the results of different studies. The Finnish National Hospital Discharge Register (NHDR) has the advantage of including the whole Finnish population. Moreover, the accuracy and coverage of the NHDR data are reported to be excellent (Mattila et al. 2008, Huttunen et al. 2014). Thus, the rates calculated from the register are not sample-based estimates but actual population results (Sund 2012).

### Pelvic fracture surgery

The annual number and incidence of pelvic fracture operations in Finland increased from 118 to 187/year and from 3.0 to 4.3 operations/100,000 person-years. This increase is most likely due to the increase in the incidence of pelvic fractures, which is most evident in the elderly population.

After 2008, the surgical treatment of pelvic fractures in Finland increased in the elderly population. This increase might be considered “minor” when compared with the increasing number and incidence of pelvic fractures. Nevertheless, new implants with anatomical design and a locking screw mechanism became available for pelvic fracture surgery during the study period and this might have had an impact on pelvic fracture operation rates.

External fixation (the Slätis frame) was used both as temporary and definitive fixation of pelvic fractures during the beginning of the study period. Since then, the role of external fixation in the treatment of pelvic fractures has diminished. One reason for this might be the increased use of pelvic binders in emergency situations. The definitive operative treatment of pelvic fractures in Finland has been centralized mainly to our 5 level I trauma centers, whereas primary stabilization and emergency surgery were previously also performed in smaller hospitals. Surgical treatment protocols of pelvic fractures have changed towards performing the definitive fixation of a pelvic fracture as a primary fracture operation after pelvic binder as the primary treatment. Therefore, the role of external fixation as a temporary fixator for the stabilization period and transport to a level I trauma center has diminished.

A limitation of this study is related to the ICD-10 coding system, which is not entirely accurate with all pelvic fractures. Codes S32.7 (multiple fractures of lumbar spine and pelvis) and S32.8 (fracture of other and unspecified parts of lumbar spine and pelvis) include both pelvic and lumbar spinal fractures, which may cause some inaccuracy in the register, and therefore the ICD-10 system is not entirely unambiguous in classifying pelvic fractures. The change in the practice of coding of the fractures might also affect the study results. The ICD-10 coding system has been used in the Finnish NHDR since 1996 while our study started in 1997. Thus, introduction of the ICD-10 coding system may have had some effect on the practice of coding at the beginning of our study period. Another limitation of this study is related to the FCP coding system, which is not entirely accurate with the codes relating to open reduction of pelvic fracture (NEJ50) and reoperation or late fracture surgery of pelvis (NEJ86). However, as the same ICD-10 and FCP coding was used during the whole study period from 1997 to 2014, this possible problem with the accuracy of the classification did not affect the reported time trends in fracture incidence. Finally, as such, the NHDR cannot separate the hospitalization events of two different pelvic fractures in a single patient. We solved this by counting one patient only once. Also, it was possible that some patients in the early study population might have had a pelvic fracture prior to the study period but been sampled into the study population merely due to re-hospitalization of the fracture. We note that this type of case must have been rare.

In summary, we observed that in Finnish adults the overall incidence of hospitalization for a pelvic fracture increased from 34 to 56/100,000 person-years between 1997 and 2014. The increase was especially apparent in low-energy fragility fractures among the elderly female population. The increasing number and incidence of pelvic fractures in the elderly population will increase the need for social and healthcare services. The main focus should be on fracture prevention.

Conception and design: VMM. Collection and assembly of data: PR. Analysis: PR. Interpretation of the data: PR, VMM, ML. Drafting of the manuscript: PR. Critical revision and final approval of the article: PR, VMM, ML, PK. 

*Acta* thanks Marianne Hansen Gillam and Alma B Pedersen for help with peer review of this study.

**Table:** Incidence of pelvic fractures (n/100,000 person-years)

	Year 1997	Year 2014
	n (95% CI)	n (95% CI)
Overall	34 (32–36)	56 (54–58)
18–64 years	14 (12–15)	19 (17–20)
≥ 65 years	121 (113–129)	169 (161–177)
Male 18–64 years	17 (15–19)	20 (18–22)
Male ≥ 65 years	57 (49–67)	100 (92–110)
Female 18–64 years	10 (8–11)	17 (15–19)
Female ≥ 65 years	159 (148–171)	220 (209–232)
